# Rare but Fatal Pasteurella multocida Infective Endocarditis: A Case Report and Literature Review

**DOI:** 10.7759/cureus.22950

**Published:** 2022-03-08

**Authors:** Mohamed Mahmoud, Khadija El Kortbi, Mohamed I Abdalla, Sheila Habib

**Affiliations:** 1 Internal Medicine, University of Texas Health Science Center at San Antonio, San Antonio, USA; 2 General Practice, Hassan II University, Faculty of Medicine, Casablanca, MAR; 3 Critical Care, University of Texas Health Science Center at San Antonio, San Antonio, USA

**Keywords:** septic emboli, heart failure, dogs, cats, septic shock, liver disease, infective endocarditis, pasteurella spp, pasteurella multocida

## Abstract

*Pasteurella multocida* is a small Gram-negative organism that usually causes a localized infection after exposure to cat or dog scratches, bites, or licking wounds. Invasive infections, such as bacteremia and endocarditis, are very rare yet serious conditions that are associated with high morbidity and mortality, particularly in patients with major comorbidities. Here, we report a case of a 47-year-old male who presented to the hospital with altered mental status two weeks after a fall and was found to have a subarachnoid hemorrhage. Further workup revealed *Pasteurella multocida *bacteremia and infective endocarditis. The patient had a complex hospital course with septic shock and acute congestive heart failure with poor clinical outcomes. A comprehensive review of the literature of all reported cases of definite *Pasteurella* endocarditis follows.

## Introduction

*Pasteurella multocida* *(P. multocida)*, a small, Gram-negative, nonmotile coccobacillus, is a normal commensal of many animals' oral flora, with cats and dogs representing the majority [1]. It can cause a variety of infections in humans, most commonly skin and soft tissue infections, following scratches, bites, or licking wounds [1]. However, invasive infections, such as bacteremia and infective endocarditis (IE), are very rare though serious conditions that are associated with high morbidity and mortality, especially in patients with major comorbidities [2]. Here, we report a case of a 47-year-old male with liver cirrhosis and diabetes found to have *P. multocida* bacteremia and endocarditis. His hospital course was complicated by septic shock, hypoxemic respiratory failure requiring intubation, septic emboli to the brain, and multiple organ failure, ultimately resulting in death.

## Case presentation

Our patient is a 47-year-old male with a history of alcohol abuse, alcoholic liver cirrhosis, hypertension, and type 2 diabetes mellitus who presented with altered mental status. Per the patient's wife, his deterioration began after a fall in the shower two weeks before presentation, followed by progressively worsening headache and multiple episodes of epistaxis. His condition continued to deteriorate and 12 hours before presentation, he developed confusion, weakness, and agitation, which prompted his presentation to the emergency department (ED).

**Figure 1:**
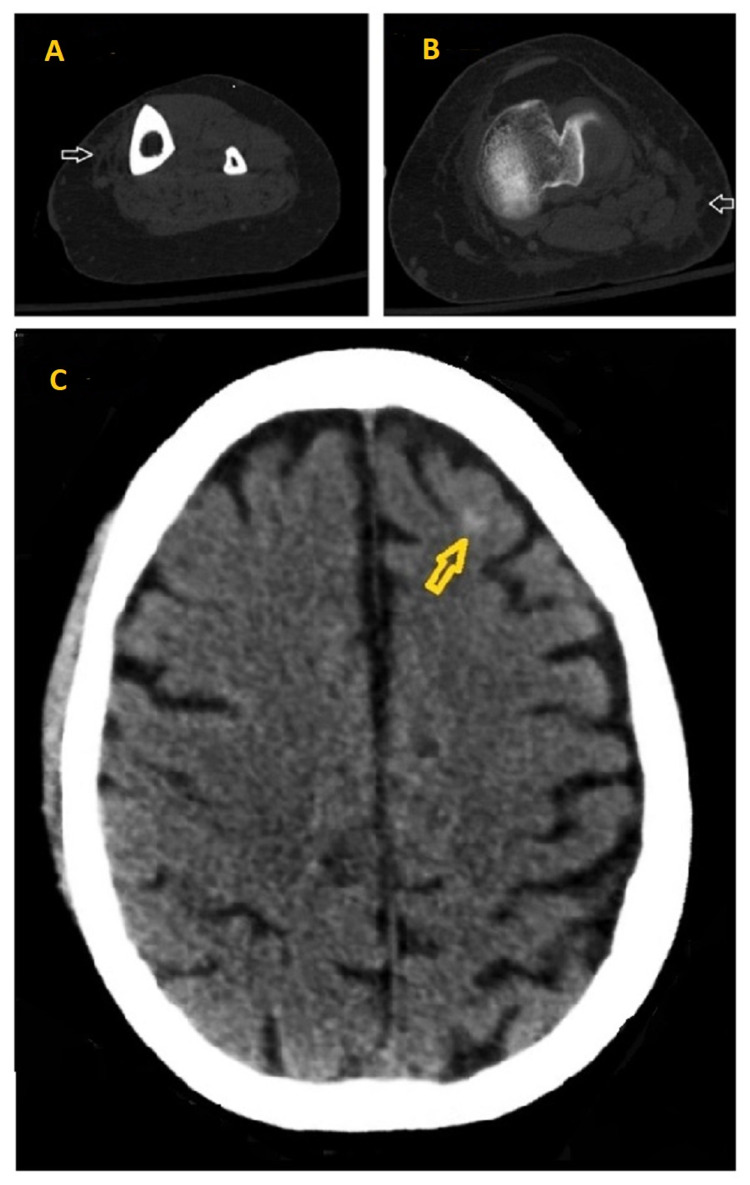
Initial CT findings 1A, 1B: CT left lower extremity showing stranding (white arrows) in the subcutaneous fat overlying the anteromedial aspect of the tibia and the posterolateral lower third of the femur respectively. 1C: CT head without IV contrast showing left frontal subarachnoid hemorrhage (yellow arrow).

In the ED, his vital signs were as follows: temperature 37.2 °C, heart rate 99/min, respiratory rate 20/min, blood pressure 115/66 mmHg, and oxygen saturation 99% on room air. On physical exam, he was ill-appearing, confused, and responding to painful stimuli only, with a Glasgow Coma Scale of 10. Skin examination was pertinent for jaundice, spider nevi, scattered ecchymoses, purpura, and scratch marks to his left lateral leg. Cardiac examination was normal except for tachycardia. Lungs were clear to auscultation, and abdominal examination was benign. The patient had +2 bilateral lower extremity pitting edema more prominent in the left side. Table 1 summarizes initial workup findings, and Figure 1 shows initial CT findings.

**Table 1:** Initial workup including labs, imaging, electrocardiogram (ECG), and 30-minute electroencephalogram (EEG) findings ALT: alanine aminotransferase, AST: aspartate aminotransferase, BNP: B-type natriuretic peptide, INR: international normalized ratio

Lab	Result	Reference Range & Units	Lab	Result	Reference Range & Units
White Blood Cell Count	23.49	(3.40-10.40) K/mcL	D-Dimer	7424	<500 ng/mL
Neutrophils Absolute	18.54	(1.50-6.60) K/mcL	Fibrinogen	437	(152-445) mg/dL
Hemoglobin	10	Male: (12.8-17.1) g/dL	C-Reactive Protein	239	(0-10) mg/L
Hematocrit	28.9	Male: (38.6-52.1) %	Lactic Acid	4.1	(0.5-2) mmol/L
Platelets	<9	(140-377) K/mcL	Ammonia	22	(11-32) mcmol/L
Sodium	127	(135-145) mmol/L	Troponin I	0.027	< 0.051 ng/mL
Potassium	3.8	(3.5-5.1) mmol/L	BNP	90	<100 pg/mL
Chloride	90	(94-106) mmol/L	Urine Analysis with Reflex Microscopy		
Blood Urea Nitrogen	102	(7-25) mg/dL	Color	Dark Yellow	Straw, Pale yellow, Dark yellow
Creatinine	4.02	Male: (0.60-1.30) mg/dL	Appearance	Cloudy	Slightly cloudy, Clear, Hazy
Glucose	20	(60-100) mg/dL	Glucose	Negative	Negative
AST	26	Male: < 35 U/L	Protein	Trace	Negative-Trace
ALT	51	Male: < 46 U/L	Bilirubin	2	Negative
Total Bilirubin	4.6	(0.2-1.2) mg/dL	Ketones	Negative	Negative
Alkaline Phosphatase	816	(45-117) U/L	Specific gravity	1025	(1001-1035)
Total Protein	5.4	(6.2-8.1) g/dL	Leukocyte esterase	1	Negative
Albumin	1.5	(3.2-5) g/dL	Blood	Negative	Negative-Trace
INR	1.5	(0.8-1.2)	Nitrites	Positive	Negative
Imaging/Test		Findings			
Chest X-ray		Bibasilar atelectasis and superior mediastinal prominence concerning for adenopathy or mass (Figure 2A).			
CT head without IV contrast		Left frontal hyperdense focus concerning for subarachnoid hemorrhage (Figure 1C) and nondisplaced right nasal bone fracture.			
CT chest without IV contrast		Trace bilateral pleural effusion with compressive atelectasis and minimally displaced anterior wedge compression deformity of the superior endplate of T3 without significant height loss.			
CT abdomen and pelvis without IV contrast		liver cirrhosis with sequelae of portal hypertension including splenomegaly and portosystemic varices, small hiatus hernia, and cholelithiasis without CT evidence of acute cholecystitis.			
CT left lower extremity without IV contrast		Stranding within the subcutaneous soft tissues predominantly along the anteromedial aspect of the tibia and the posterolateral lower third of the femur is most suggestive of cellulitis (Figures 1A, 1B).			
Bilateral lower extremity Doppler ultrasound		Negative for deep venous thrombosis.			
ECG		Normal sinus rhythm with premature atrial complexes, left axis deviation, and incomplete left bundle branch block.			
30 minutes EEG		Evidence of mild to moderate encephalopathy with no seizure activity.			

**Figure 2:**
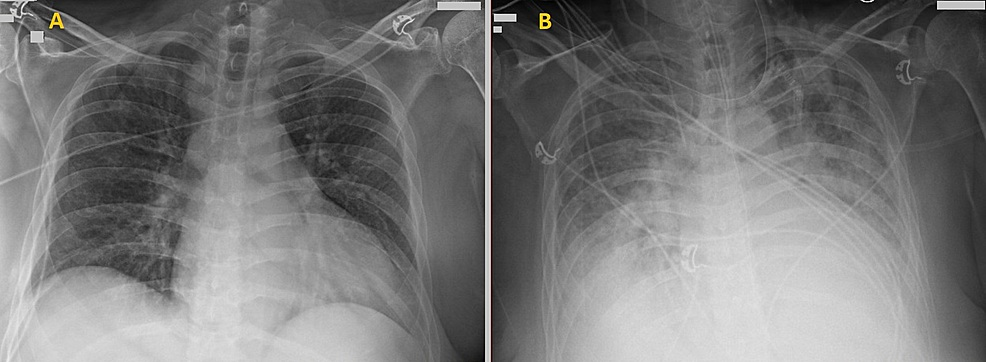
Chest X-ray on admission and two days later 2A: Chest X-ray on admission showing bibasilar atelectasis and superior mediastinal prominence concerning for adenopathy or mass. Two days later (Figure 2B), new moderate pulmonary edema developed.

The patient received intravenous (IV) fluids and platelet transfusion, resulting in improved creatinine to 3.31 mg/dL, platelets to 22 K/mcL, and lactic acid to 1.8 mmol/L. He was then admitted to the Medical Intensive Care Unit (MICU) and started on IV vancomycin and cefepime for sepsis. Two sets of blood cultures grew Gram-negative rods 11 hours after collection. Identification of *P. multocida *was done using* *matrix-assisted laser desorption/ionization time-of-flight mass spectrometry (MALDI-TOF MS). The disk diffusion test revealed susceptibility to penicillin, ampicillin, ceftriaxone, and levofloxacin. Urine culture was negative for any bacterial growth. Further questioning of the patient's wife revealed a history of scratch by an outdoor cat to his left leg two weeks before presentation.

**Figure 3:**
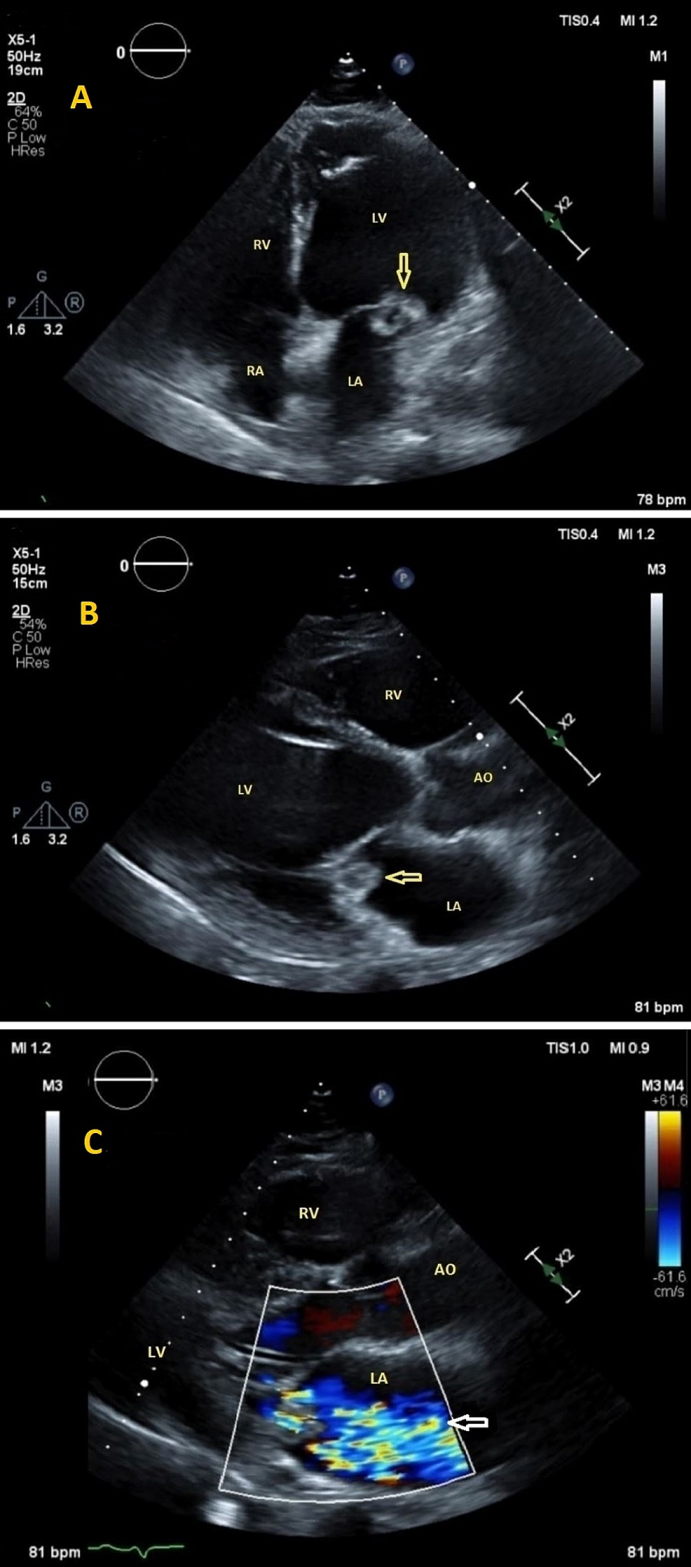
Transthoracic echocardiography 3A (apical 4 chamber view) and 3B (parasternal long-axis view) show a rounded irregular mass with a lucent center attached to the posterior leaflet of the mitral valve consistent with vegetation (yellow arrows), and a severely dilated left ventricle. 3C: Color Doppler showing severe mitral regurgitation (white arrow).

Two days later, the patient developed tachypnea, tachycardia, and increased work of breathing. Chest X-ray showed increased infiltrates concerning for moderate pulmonary edema (Figure 2B) that was not found on the initial chest X-ray (Figure 2A). He was intubated and started on vasopressors for septic shock. Transthoracic echocardiogram (TTE) revealed a 1.5 cm x 1.3 cm vegetation attached to the posterior leaflet of the mitral valve (Figures 3A, 3B), severe mitral regurgitation (MR) (Figure 3C), and an estimated left ventricular ejection fraction (LVEF) of 61%.

**Figure 4:**
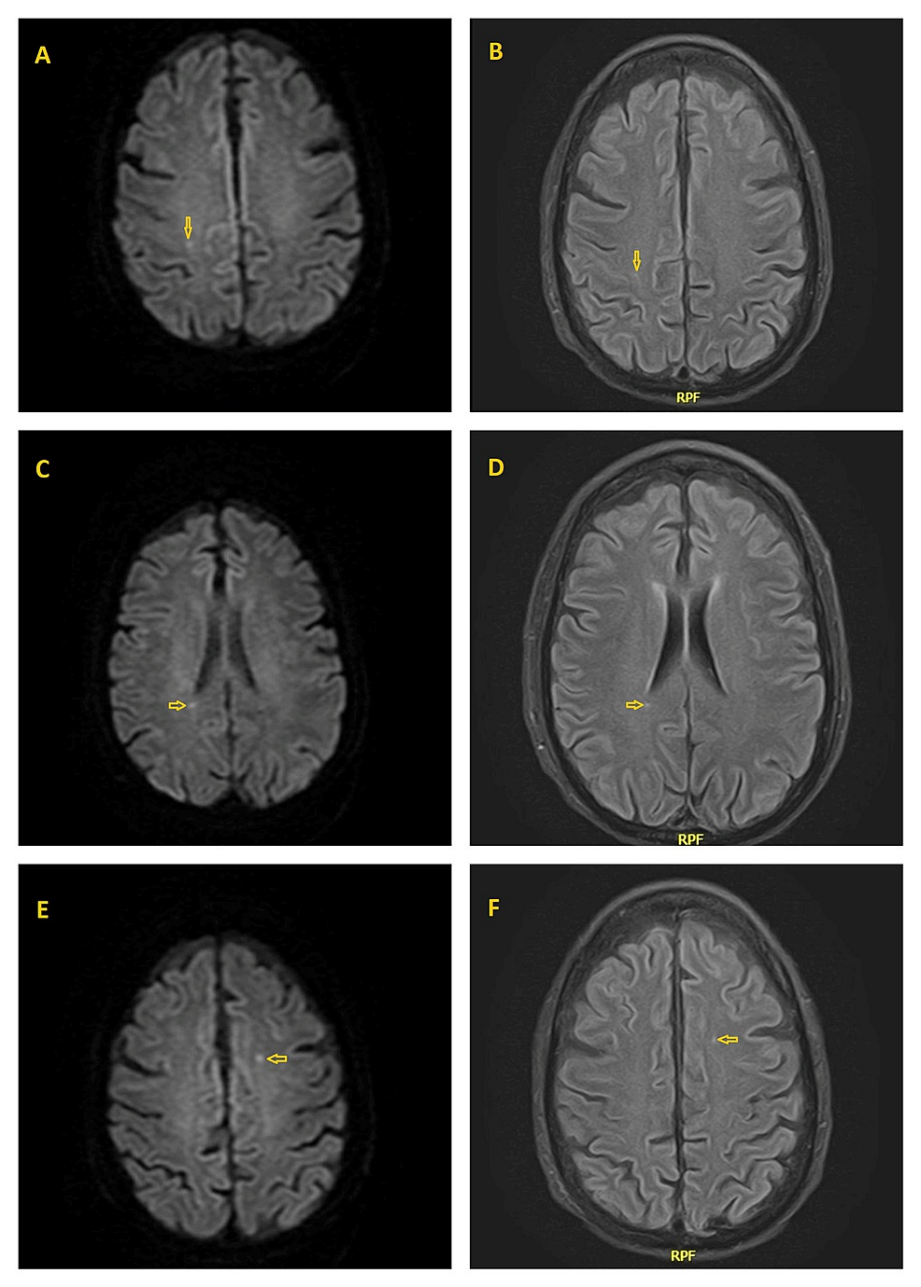
Magnetic resonance imaging (MRI) of the brain Diffusion-weighted MRI (4A, 4C, 4E) and fluid-attenuated inversion recovery (FLAIR) MRI (4B, 4D, 4F) of the brain showing subacute lacunar stroke (yellow arrows) with distribution suggestive of embolic phenomena.

Repeat blood culture the next day was negative. Antibiotics were switched to IV ceftriaxone 2 gm q12hr and IV metronidazole 500 mg q8hr to cover for possible meningitis and aspiration pneumonia given his worsening mentation. Four days after admission, brain MRI showed signs of subacute lacunar infarcts with distribution suggestive of embolic phenomena (Figure 4). Spine MRI was negative for osteomyelitis, discitis, or spinal abscess. Cardiothoracic surgery was consulted and recommended repeating a TTE in one week. The patient was deemed a poor surgical candidate for surgical mitral valve replacement given his high MELD Na of 29, Child C liver cirrhosis, and subarachnoid hemorrhage. 

Three days later, the patient was extubated, following commands, and off vasopressors. He was then transferred to the medicine floor. However, after a few days, he developed severe pulmonary edema requiring bilevel positive airway pressure (BiPAP). Furthermore, he developed intermittent fever and anemia requiring blood transfusion. Physical exam was pertinent for diffuse anasarca, a blowing systolic murmur best heard at the lower left sternal border, and diffuse crackles bilaterally. B-type natriuretic peptide (BNP) was 1200 pg/mL and troponin I 0.22 ng/mL. ECG showed sinus tachycardia and was negative for acute ischemic changes. CT pulmonary embolism (PE) study was negative for PE but showed marked worsening bilateral infiltrates, bilateral moderate to large pleural effusions, and right heart chamber predominant cardiomegaly. The patient was started on bumetanide drip 1 mg/hr and antibiotics were switched to IV vancomycin and piperacillin-tazobactam to cover for healthcare/ventilator-associated pneumonia. He was then transferred to the MICU and was re-intubated.

A repeat TTE revealed an increase in the size of mitral valve vegetation to 2.2 cm x 1.5 cm. The MICU course was complicated by anemia and hemoptysis concerning for diffuse alveolar hemorrhage requiring blood transfusion; however, while receiving blood, the patient developed worsening oxygenation likely due to severe MR caused by the enlarging vegetation and volume overload. Blood transfusion was held, and the patient’s diuretic regimen was increased. 

Unfortunately, the patient’s mental status did not improve and given his multiple comorbidities precluding him from aggressive interventions and prolonged complicated hospital course, his family decided to pursue comfort measures. He was palliatively extubated and transferred to the inpatient hospice service where he eventually passed comfortably.

## Discussion

**Figure 5:**
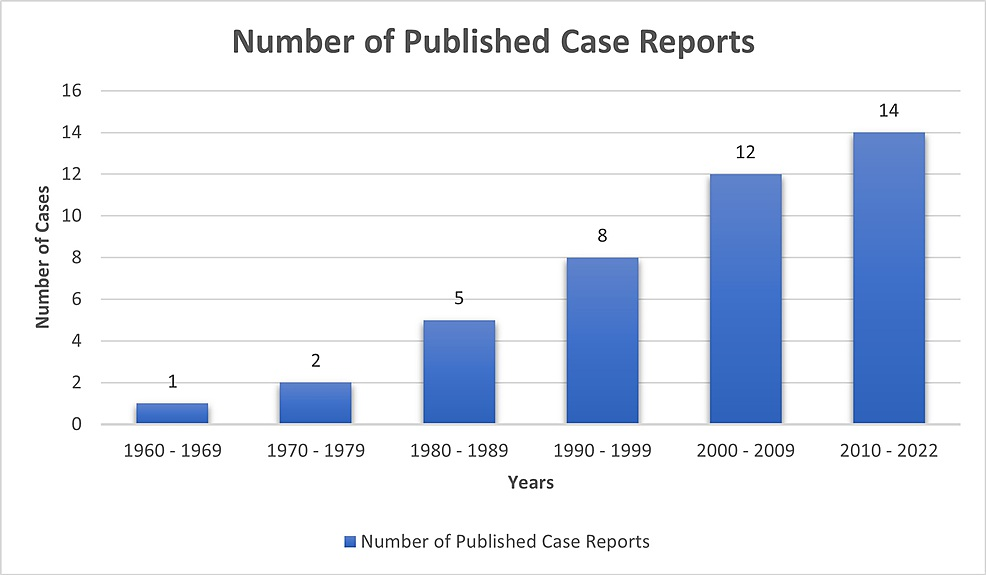
A chart showing the increasing number of case reports from 1960 to present

*Pasteurella* species (spp) are normal commensals of many animals' oral bacterial flora, primarily cats and dogs [1]. Human infection by *Pasteurella* usually causes localized skin and soft tissue infections [1]. However, infective endocarditis is rare, with only 42 cases, including ours, reported in the literature. A PubMed search was conducted using keywords "*Pasteurella*" and "endocarditis". Only cases that met the Modified Duke infective endocarditis criteria [3] and with microbiologically proven *Pasteurella* infection were included and reviewed manually. Further studies were identified from the references of the selected cases. The number of reported cases has been increasing, especially in the last two decades (Figure 5). Males were affected more than females, comprising 62% of the reported cases. The mean age was 56.7 ± 16.5, ranging from 17 to 88 years. Table 2 summarizes the literature review of all reported cases of definite *Pasteurella* endocarditis. Table 3 shows a detailed review of all cases.

**Table 2:** Summary of literature review of all reported cases of definite Pasteurella infective endocarditis IVD: intravenous drugs, P: Pasteurella, PMH: past medical history, SD: standard deviation, Spp: species

Variables	Number of Cases (%) N=42
		Age (mean ± SD) years	(56.7 ± 16.5)
< 35 years	3 (7%)
35-70 years	10 (24%)
>70 years	29 (69%)
Gender	
Male	26 (62%)
Female	15 (36%)
Unspecified	1 (2%)
Location	
USA	15 (36%)
France	9 (21%)
Japan	4 (10%)
UK	3 (7%)
Saudi Arabia	2 (5%)
Others (countries that reported 1 case only)	9 (21%)
Risk Factors	
Substance Abuse (Smoking, Alcohol, or IVD)	10 (24%)
Liver Disease	7 (17%)
Heart Disease	20 (48%)
Previous Endocarditis	4 (10%)
Diabetes Mellitus	2 (5%)
Solid Organ Transplant	1 (2%)
Immunosuppressive Therapy	1 (2%)
Cancer	1 (2%)
Healthy With No PMH	6 (14%)
Pasteurella Spp	
Multocida	29 (69%)
Non-Multocida Spp	11 (26%)
P. Haemolytica	3 (7%)
P. Dagmatis	3 (7%)
P. Pneumotropica	3 (7%)
P. Ureae	2 (5%)
Unspecified	2 (5%)
Exposure	
Exposed to Animals	31 (74%)
Cats, Dogs, or Both	29 (69%)
Sheep	2 (5%)
Fish	1 (2%)
Known Bite, Scratch, or Lick	15 (36%)
Not Exposed	7 (17%)
Unknown	4 (10%)
Valve affected	
Native Valve	35 (83%)
Mitral	16 (38%)
Aortic	14 (33%)
Tricuspid	4 (10%)
Pulmonary	1 (2%)
Prosthetic Valve	8 (19%)
Aortic	7 (17%)
Mitral	1 (2%)
Unknown	1 (2%)
Dual valve affected	2 (5%)
Native Mitral and Tricuspid	1 (2%)
Native Tricuspid and Aortic prosthesis	1 (2%)
Treatment	
Antibiotics	41 (98%)
Surgery	18 (43%)
Outcome	
Survived	29 (69%)
Died	11 (26%)
Unspecified	2 (5%)

**Table 3:** Detailed summary of all case reports of Pasteurella endocarditis IE: infective endocarditis, DM: diabetes mellitus, Spp: species, CHF: congestive heart failure, UK: United Kingdom, USA: United States of America, NW: Northwest, *: This case represents the currently presented case. "Cured" in the outcome is defined as full recovery.

Case	1^st^ Author	Age	sex	Year of publication	Country	Reported liver disease	Reported heart disease	Previous IE	DM	Current substance abuse	Transplant/ cancer/ immunosuppresive therapy	Exposure	Pasteurella Spp	Antibiotics	Resistance	Valve	Surgery	Outcome
1	Porter [2]	66	F	2020	USA	No	Yes	No	No	No	No	Cat	Multocida	Yes	Unknown	Aortic Prosthesis & Tricuspid	Yes	Cured
2	Al-Ghonaim [4]	50	M	2006	Saudi Arabia	No	No	No	No	No	No	Sheep	Multocida	Yes	Unknown	Aortic	Yes	Cured
3	Tirmizi [5]	34	M	2012	USA	No	Yes	No	No	No	No	Fish, sheep	Pneumotropica	Yes	Unknown	Tricuspid	No	Cured
4	Brass [6]	40	M	1983	USA	No	No	No	No	Yes	No	Unknown	Ureae	Yes	No	Mitral, Tricuspid	No	Went into coma
5	Ahlsson [7]	70	M	2016	Sweden	No	No	No	No	Yes	No	Cat/ bite - scratch	Multocida	Yes	Unknown	Aortic	Yes	Cured
6	Branch [8]	50	M	2015	Japan	No	No	No	No	No	No	None	Multocida	Yes	Piperacillin-Tazobactam	Mitral	Yes	Cured
7	Kollu [9]	57	F	2021	USA	No	Yes	Yes	No	No	No	Dog/ lick - bite	Unspecified	Yes	No	Prosthetic Mitral	yes	Cured
8	Camou [10]	79	F	2005	France	Yes	No	No	No	No	No	Cat/ bite	Multocida	Yes	Unknown	Mitral	No	Died
9	Camou [10]	81	F	2005	France	Yes	Yes	No	No	No	No	Cat	Multocida	Yes	Unknown	Aortic prosthesis	No	Died of septic shock with Burkholderia cepacia bacteremia and Candida fungemia.
10	Sauvet [11]	78	F	2004	France	No	No	No	No	Yes	No	Unknown	Multocida	Yes	Unknown	Aortic	planned	Developed CHF, Surgery planned
11	Guilbart [12]	74	M	2015	France	No	Yes	No	No	Yes	No	Dog/ licked wound	Multocida	Yes	lincosamides and aminoglycosides	Mitral	No	Died
12	Hombal [13]	61	M	1992	USA	No	Yes	No	No	No	No	Dog/ licked leg ulcer	Multocida	Yes	Unknown	Aortic	No	Died
13	Thamlikitkul [14]	17	M	1990	Thailand	Yes	Yes	No	No	No	No	None	Multocida	Yes	Unknown	Mitral	No	Died
14	Sorbello [15]	55	F	1994	USA	Yes	No	No	No	Yes	No	Cat/ bite and scratch	Dagmatis	Yes	Unknown	Mitral	No	Cured
15	Carter [16]	79	F	2021	UK	No	Yes	No	Yes	No	Yes	Cat/ scratch	multocida	Yes	No	Mitral	No	Died
16	Guerin [17]	62	F	1980	France	Yes	No	No	No	Yes	No	Cat/ bite	Multocida	No	Unknown	Aortic	No	Died
17	Satta [18]	38	M	2012	UK	No	Yes	No	No	No	Yes	Cat-Dog	Multocida	Yes	Unknown	Aortic prothesis	No	Cured
18	Abad [19]	37	M	2003	NW Africa	No	No	No	No	No	No	Unknown	Multocida	Yes	penicillin	Mitral	Yes	Cured
19	Reinsch [20]	66	M	2008	Germany	No	Yes	Yes	No	Yes	No	Cat/ bite	Multocida	Yes	Unknown	Aortic Prosthesis	Yes	Died from septic shock after surgery due to Pseudomonas endocarditis following a dental procedure
20	Doty [21]	54	F	1963	USA	No	Yes	No	No	No	No	Household Pets	Haemolytica	Yes	Bacitracin and Triacetylo-leandomycin	Mitral	No	Died
21	Gump [22]	44	M	1972	USA	No	Yes	No	No	No	No	Cat /bite	New species	Yes	Unknown	Unknown	No	Cured
22	Lehmann [23]	51	M	1977	Norway	No	No	No	No	No	No	None	Multocida atypical strain	Yes	Streptomycin, lincomycin, Cephalosporins, Sulpha	Aortic	Yes	Cured
23	Singh [24]	50	M	1983	UK	No	No	No	No	Yes	No	Dog	Multocida	Yes	No	Mitral	no	Cured
24	Cornaert [25]	33	F	1987	France	No	No	No	No	No	No	Unknown	Pneumotropica	Yes	Unknown	Mitral	Yes	Cured
25	Salmon [26]	63	F	1989	France	No	No	No	No	No	No	Dog	Multocida	Yes	Unknown	Aortic	Yes	Cured
26	Yaneza [27]	40	M	1991	Saudi Arabia	No	No	No	No	No	No	None	Haemolytica	Yes	No	Aortic	No	Died
27	Yamamoto [28]	59	M	1993	Japan	No	Yes	Yes	No	No	No	None	Ureae	Yes	No	Mitral	No	Cured
28	Genné [29]	38	F	1996	Switzerland	Yes	No	No	No	Yes	No	Cat	Multocida	Yes)	Unknown	Aortic	No	Cured
29	Nettles [30]	72	F	1997	USA	No	Yes	No	No	No	No	Cat	Multocida	Yes	Unknown	Aortic Prosthesis	No	Cured
30	Vasquez [31]	65	M	1998	USA	Yes	Yes	No	No	No	No	Cat-Dog/ lick	Multocida	Yes	Unknown	Aortic	No	Died
31	Rosenbach [32]	78	M	2001	USA	no	Yes	No	No	No	No	None	Dagmatis	Yes	Unknown	Aortic prosthesis	No	Cured
32	Fukumoto [33]	48	M	2002	Japan	No	Yes	No	No	No	No	Dog	Multocida	Yes	Unknown	Mitral	Yes	Cured
33	Fayad [34]	48	M	2003	France	Yes	No	No	No	No	No	Dog	Multocida	Yes	Unknown	Aortic	Yes	Cured
34	Dan [35]	43	?	2005	Romania	No	Yes	No	No	No	No	Pets/ scratch	Pneumotropica	Yes	Unknown	Mitral	Yes	Cured
35	Graf [36]	36	M	2007	Austria	No	No	No	No	Yes	No	Cat-Dog	Multocida	Yes	Unknown	Pulmonary	Yes	Cured
36	Naba [37]	88	F	2009	Lebanon	No	No	No	No	No	No	Cat/ bite	Multocida	Yes	Unknown	Tricuspid	No	Cured
37	Khan [38]	82	M	2012	USA	No	Yes	No	No	No	No	Cat	Multocida	Yes	Unknown	Aortic	No	Cured
38	Strahm [39]	77	M	2012	USA	No	Yes	Yes	No	No	No	Cat/ lick	Dagmatis	Yes	Unknown	Aortic prosthesis	Yes	Cured
39	Fayad [40]	62	M	2012	France	No	No	No	No	No	No	Dog	Haemolytica	Yes	Unknown	Aortic	Yes	Cured
40	Mikaberidz [41]	60	F	2013	USA	No	No	No	No	No	No	Cat-Dog	Multocida	Yes	Unknown	Aortic	Yes	Cured
41	Yuji [42]	50	M	2015	Japan	No	No	No	No	No	No	None	Multocida	Yes	Unknown	Mitral	Yes	Cured
42	Mahmoud*	47	M	2022	USA	Yes	No	No	Yes	Yes	No	Cat/ scratch	Multocida	Yes	No	Mitral	No	Died

Our case satisfied the Modified Duke criteria for the diagnosis of infective endocarditis [3]; one major criterion: evidence of endocardial involvement, and three minor criteria: fever, blood culture, and vascular phenomena (septic emboli to the brain).

Of 31 cases (74%) who reported exposure to animals, 29 (94%) had exposure to cats, dogs, or both. One had exposure to sheep [4], and another was exposed to fish and sheep [5]. Fifteen (48%) had a known history of scratches, bites, or licking non-intact skin. Of all reported cases, seven (17%) had no history of animal exposure. *P. multocida* has been isolated from the respiratory tract of healthy individuals who have frequent exposure to animals [1].

*P. multocida* was the most reported spp; 29 cases (69%). 11 cases (26%) had infection with non-multocida spp, including *P. haemolytica* (3), *P. dagmatis* (3). *P. pneumotropica* (3), and *P. ureae* (2).

The native mitral valve was the most affected (16 cases), followed by native aortic (14), prosthetic aortic (7), native tricuspid (4), native pulmonary (1), and prosthetic mitral (1) valves. Two cases reported dual valve infection [2,6].

Risk factors included liver disease in seven (17%), heart disease in 20 (48%), prior endocarditis in four (10%), substance abuse in 10 (24%), diabetes mellitus in two (5%), solid organ transplant/immunosuppressive therapy in one (2%), and malignancy in one (2%). However, *Pasteurella* IE has also been reported in six (14%) healthy individuals. Immunocompromised patients are at higher risk of severe disease and complications such as sepsis, septic shock, and multiorgan failure [2,43]. A comprehensive review of 119 cases of *P. multocida* bacteremia reported comorbid conditions, such as chronic liver disease, diabetes mellitus, malignancy, and immunosuppressive therapy in 67% of patients, and the mortality rate was 31% at 30 days [43]. On multivariate analysis, having major comorbid conditions was the only factor associated with mortality (OR 2.78, 95% CI 1.01-7.70: P-value 0.04) [43].

The overall mortality rate in previously reported cases of *Pasteurella* endocarditis was 26% (11 cases), of which 64% (7 cases) had major comorbidities. A recent analysis of 32 cases of Pasteurella endocarditis demonstrated a statistically significant association between comorbid liver disease and mortality rate despite the low number of cases [2].

Our patient had a complex hospital course consisting of sepsis, septic shock requiring vasopressors, and acute hypoxemic respiratory failure secondary to cardiogenic pulmonary edema due to severe mitral regurgitation requiring intubation, resulting in death. Diabetes mellitus and liver cirrhosis were the predisposing conditions that led to severe invasive infection in this case.

Complications are not uncommon and related to bacteremia and valve vegetation. Reported complications included sepsis, septic emboli [8-9], congestive heart failure [2,10-11], septic shock [2,10,12-13,31], mycotic aneurysm [9,14], intracranial hemorrhage [14-15], septic arthritis [12,16], and osteomyelitis [15]. One case reported rhabdomyolysis and hearing loss [41].

There are no clear guidelines for treating *Pasteurella* endocarditis, and data are limited to a small number of case reports. All patients received antibiotics except one who died shortly after presentation [17]. *Pasteurella* spp is often susceptible to penicillin [13,18]. Broad-spectrum cephalosporins, piperacillin-tazobactam, and ampicillin-sulbactam can be used alternatively. One case of *Pasteurella* endocarditis of the prosthetic mitral valve reported successful treatment with six weeks of IV penicillin without surgery [9]. Tirmizi et al. reported successful treatment of *P. pneumotropica* endocarditis of the native tricuspid valve with six weeks of oral ciprofloxacin [5]. One case of *P. multocida* endocarditis of the aortic valve prosthesis had a penicillin allergy and was successfully treated with six weeks of IV ceftriaxone [18]. Two cases reported resistance to penicillin and piperacillin-tazobactam, respectively [8,19]. Duration of treatment was variable among reported cases, and it depended on the severity, course of the disease, co-existing conditions, and antibiotic susceptibility. Carter et al. recommended initial six weeks of antibiotics therapy based on the clinical response and the average duration in previously reported patients who were successfully treated with antibiotics only [16]. Of all 29 cases (69%) who survived, 12 (41 %) received antibiotics only without surgery.

Of all reported cases, 18 (43%) required surgery. Indications for surgical intervention were severe valvular insufficiency, persistent symptoms despite antibiotic therapy, and aortic root abscess. Seventeen out of 18 (94%) cases who underwent surgery were cured while only one patient died four months after discharge due to septic shock secondary to *Pseudomonas* endocarditis of the native mitral and prosthetic aortic valves four weeks after a dental procedure [20]. Porter et al. suggested that surgery should be offered to all patients who have no absolute contraindications given the cure rate of 100% after surgical valve replacement [2]; however, their analysis did not consider the severity of illness, medical comorbidities, and indications for surgical intervention [16]. Moreover, they did not include the patient who died four months after surgery from *Pseudomonas* endocarditis [20]. Carter et al. suggested surgical intervention only if there is any indication, as with any case of other bacterial endocarditis [16]. The same study reported a patient with *P. multocida* endocarditis and septic arthritis successfully treated with antibiotics only despite his comorbidities.

There is no doubt that *Pasteurella* endocarditis is a rapidly progressive disease and is associated with high morbidity and mortality. It took approximately two weeks for our patient to develop complications following a cat scratch, one week from presentation to develop an increase in the valve vegetation size, and four weeks from presentation to death. He received a total duration of three weeks of antimicrobial therapy, but unfortunately, he was a poor surgical candidate due to significant comorbidities.

Due to its rarity, it is hard to conclude the proper management of *Pasteurella* endocarditis from the current literature. In the meantime, early recognition of the disease, interval echocardiograms to assess the vegetation size and possible complications, IV antibiotics, and early source control with surgical valve replacement for patients who have indications are the mainstay of treatment.

## Conclusions

Infective endocarditis caused by *Pasteurella* spp is a rare though potentially serious and rapidly progressive disease with only 42 cases reported in the literature. It carries a high risk of morbidity and mortality, particularly in patients with comorbid conditions such as liver disease. Treatment is typically IV antibiotics, and surgery for source control should be considered on a case-by-case basis. Due to its rarity, further research is required to study the nature of disease progression, determine the appropriate duration of antimicrobial therapy, and identify the average time from symptom onset to surgery and its correlation with clinical outcomes.

## References

[1] Weber DJ, Wolfson JS, Swartz MN, Hooper DC (1984). Pasteurella multocida infections. Report of 34 cases and review of the literature. Medicine (Baltimore).

[2] Porter RS, Hay CM (2020). Pasteurella endocarditis: a case report and statistical analysis of the literature. Case Rep Infect Dis.

[3] Li JS, Sexton DJ, Mick N (2000). Proposed modifications to the Duke criteria for the diagnosis of infective endocarditis. Clin Infect Dis.

[4] Al-Ghonaim MA, Abba AA, Al-Nozha M (2006). Endocarditis caused by Pasteurella multocida. Ann Saudi Med.

[5] Tirmizi A, Butt S, Molitorisz S (2012). First reported case of Pasteurella pneumotropica tricuspid valve endocarditis. Int J Cardiol.

[6] Brass EP, Wray LM, McDuff T (1983). Pasteurella ureae meningitis associated with endocarditis. Report of a case and review of the literature. Eur Neurol.

[7] Ahlsson A, Friberg Ö, Källman J (2016). An angry cat causing Pasteurella multocida endocarditis and aortic valve replacement—a case report. Int J Surg Case Rep.

[8] Branch J, Kakutani T, Kuroda S, Shiba Y, Kitagawa I (2015). Pasteurella multocida infective endocarditis: a possible link with primary upper respiratory tract infection. Intern Med.

[9] Kollu VS, Archibald L, Edwards M, Janelle JW, Hong KW, Kalyatanda G (2021). Pasteurella cerebral mycotic aneurysm: a case report and review of the literature. Cureus.

[10] Camou F, Guisset O, Pereyre S, Gabinski C, Viallard JF, Mercié P, Pellegrin JL (2005). Endocarditis due to Pasteurella sp. Two cases [Article in French]. Med Mal Infect.

[11] Sauvet F, Graffin B, Cremades S, Chemsi M, Leyral G, Paris JF, Carli P (2004). Pasteurella multocida endocarditis revealed by inflammatory rachialgia [Article in French]. Rev Med Interne.

[12] Guilbart M, Zogheib E, Hchikat AH (2015). Fatal multifocal Pasteurella multocida infection: a case report. BMC Res Notes.

[13] Hombal SM, Dincsoy HP (1992). Pasteurella multocida endocarditis. Am J Clin Pathol.

[14] Thamlikitkul V, Sangruchi T (1990). Pasteurella multocida infective endocarditis: a case report. J Med Assoc Thai.

[15] Sorbello AF, O'Donnell J, Kaiser-Smith J, Fitzharris J, Shinkarow J, Doneson S (1994). Infective endocarditis due to Pasteurella dagmatis: case report and review. Clin Infect Dis.

[16] Carter E, Iroegbu U, Baig W, Sandoe JA (2021). Pasteurella multocida endocarditis with septic arthritis: case report and review of the literature. EMJ Microbiol Infect Dis.

[17] Guerain JM, Segrestaa JM, Lamotte M (1980). Pasteurella multocida endocarditis [Article in French]. Nouv Presse Med.

[18] Satta G, Gorton RL, Kandil H (2012). Prosthetic valve endocarditis caused by Pasteurella in a penicillin allergic patient: challenges in diagnosis and treatment. Infect Dis Rep.

[19] Abad C, Cáceres JJ, Ferrer JM, González L, Alvarez F, Bordes A (2003). Pasteurella multocida empyema in a patient after mitral valve replacement secondary to endocarditis [Article in Spanish]. An Med Interna.

[20] Reinsch N, Plicht B, Lind A (2008). Recurrent infective endocarditis with uncommon Gram-negative Pasteurella multocida and Pseudomonas aeruginosa: a case report. J Heart Valve Dis.

[21] Doty GL, Loomus GN, Wolf PL (1963). Pasteurella endocarditis. N Engl J Med.

[22] Gump DW, Holden RA (1972). Endocarditis caused by a new species of Pasteurella. Ann Intern Med.

[23] Lehmann V, Knutsen SB, Ragnhildstveit E, Skagseth E, Solberg CO (1977). Endocarditis caused by Pasteurella multocida. Scand J Infect Dis.

[24] Singh CP, Spurrell JR (1983). Pasteurella multocida endocarditis. Br Med J (Clin Res Ed).

[25] Cornaert P, Masson P, Forzy G, Graux P, Camblin J, Dutoit A, Croccel L (1987). Infectious endocarditis caused by rare germs. Review of the literature apropos of 2 cases [Article in French]. Ann Cardiol Angeiol (Paris).

[26] Salmon D, Fantin B, Bricaire F, Vilde JL, Pangon B, Ferand D (1989). Endocarditis due to Pasteurella multocida with glomerulonephritis. Am J Med.

[27] Yaneza AL, Jivan H, Kumari P, Togoo MS (1991). Pasteurella haemolytica endocarditis. J Infect.

[28] Yamamoto K, Ikeda U, Ogawa C, Fukazawa H, Eto M, Shimada K (1993). Pasteurella ureae endocarditis. Intern Med.

[29] Genne D, Siegrist HH, Monnier P, Nobel M, Humair L, de Torrente A (1996). Pasteurella multocida endocarditis: report of a case and review of the literature. Scand J Infect Dis.

[30] Nettles RE, Sexton DJ (1997). Pasteurella multocida prosthetic valve endocarditis: case report and review. Clin Infect Dis.

[31] Vasquez JE, Ferguson DA Jr, Bin-Sagheer S, Myers JW, Ramsak A, Wilson MA, Sarubbi FA (1998). Pasteurella multocida endocarditis: a molecular epidemiological study. Clin Infect Dis.

[32] Rosenbach KA, Poblete J, Larkin I (2001). Prosthetic valve endocarditis caused by Pasteurella dagmatis. South Med J.

[33] Fukumoto Y, Moriyama Y, Iguro Y, Toda R, Taira A (2002). Pasteurella multocida endocarditis: report of a case. Surg Today.

[34] Fayad G, Modine T, Mokhtari S (2003). Pasteurella multocida aortic valve endocarditis: case report and literature review. J Heart Valve Dis.

[35] Dan M, Prisacariu C, Georgescu GI, Georgescu-Arsenescu C, Tinică G, Buiuc D (2005). Subacute bacterial endocarditis due to Pasteurella pneumotropica. Case report [Article in Romanian]. Rev Med Chir Soc Med Nat Iasi.

[36] Graf S, Binder T, Heger M, Apfalter P, Simon N, Winkler S (2007). Isolated endocarditis of the pulmonary valve caused by Pasteurella multocida. Infection.

[37] Naba MR, Araj GF, Kanafani ZA, Kanj SS (2009). First case of Pasteurella multocida endocarditis of the tricuspid valve: a favorable outcome following medical treatment. Int J Infect Dis.

[38] Khan MF, Movahed MR, Jung J (2012). Pasteurella multocida endocarditis. J Heart Valve Dis.

[39] Strahm C, Goldenberger D, Gutmann M, Kuhnert P, Graber P (2012). Prosthetic valve endocarditis caused by a Pasteurella dagmatis-like isolate originating from a patient's cat. J Clin Microbiol.

[40] Fayad G, Modine T, Koussa M, Senneville E, Leroy O (2012). First documented surgical case of human aortic valve endocarditis caused by Pasteurella haemolytica. J Heart Valve Dis.

[41] Mikaberidz N, Li EY, Taub CC (2013). Pasteurella multocida infective endocarditis in an immunocompetent patient complicated by rhabdomyolysis and permanent hearing loss. J Cardiovasc Dis Res.

[42] Yuji D, Tanaka M, Katayama I, Noguchi K (2015). Pasteurella multocida infective endocarditis. J Heart Valve Dis.

[43] Chatelier E, Mahieu R, Hamel JF (2020). Pasteurella bacteraemia: impact of comorbidities on outcome, based on a case series and literature review. Int J Infect Dis.

